# Following laser induced changes of plant phenylpropanoids by Raman microscopy

**DOI:** 10.1038/s41598-018-30096-3

**Published:** 2018-08-07

**Authors:** Batirtze Prats-Mateu, Peter Bock, Martina Schroffenegger, José Luis Toca-Herrera, Notburga Gierlinger

**Affiliations:** 1 Institute for Biophysics, Department of Nanobiotechnology, BOKU-University of Natural Resources and Life Sciences, Muthgasse 11/II, 1190 Vienna, Austria; 2 Institute of Biologically inspired materials, Department of Nanobiotechnology, BOKU-University of Natural Resources and Life Sciences, Muthgasse 11/II, 1190 Vienna, Austria

## Abstract

Raman microscopy is a powerful imaging technique for biological materials providing information about chemistry in context with microstructure. A 532 nm laser is often used as excitation source, because high spatial resolution and signal intensity can be achieved. The latter can be controlled by laser power and integration time, whereby high power and long times give good signal to noise ratio. However, most biological materials absorb in the VIS range and fluorescence masking the signal or even sample degradation might be hindering. Here, we show that on lignified plant cell walls even very short integration times and low laser powers induce a change in the ratio of the lignin bands at 1660 and 1600 cm^−1^. Time series on lignin model compounds revealed this change only in aromatic molecules with two OH-groups, such as coniferyl alcohol. Therefore, we conclude that monolignols are present in the cell wall and responsible for the observed effect. The solvent selectivity of the changes points to a laser induced polymerization process. The results emphasize how crucial careful adjustment of experimental parameters in Raman imaging of biological materials is and show the potential of time series and repeated imaging to get additional insights (*e.g*. monolignols).

## Introduction

Phenolic compounds in plants comprise a wide range of molecules with several functions including signaling^1^, disease resistance^2^, defense^3^, wound healing^4^, mechanical stability^5,6^ and waterproofing for water transport^7,8^. In the plant cell wall, 4-vinylphenols called monolignols polymerize into lignin and are, together with hemicelluloses, the embedding matrix for cellulose microfibrils^9,10^. Lignin is a very complex polymer, with several different linkages present^11,12^ that generate lignocellulosic biomass recalcitrance, *e.g*. in pulp or biofuel industry. The number of studies on the biosynthesis, structure, deposition and use of this polymer have enormously increased over the years *e.g*.^13–20^. First of all, to know how the natural lignification occurs *in vivo* and also to overcome the recalcitrance issue^21^.

Marker-free techniques such as UV absorption and fluorescence microscopy that profit of the lignin auto-fluorescence have been used in the past decades to study the lignin polymer^22–25^. Although these techniques are very useful in monitoring lignin, they often need narrow excitation and emission filters to discriminate between specific aromatic compounds. Raman imaging has become a well-stablished experimental technique for chemical analysis of a large range of materials. The Raman Effect allows recording the molecular fingerprint of the sample. Information on all chemical groups is gained at once without taking multiple images on the same sample, as in fluorescence microscopy. Furthermore, the possibility of applying this method in aqueous native conditions makes it very attractive for the characterization of biological samples^26^ and plenty of successful examples are available today in the literature^27–33^, including wood^34,35^, plants^36,37^ and studies on the aromatic lignin^38–41^. The lateral resolution of any optical system, including Raman imaging, is determined by the diffraction limit of light (*r*_*xy*_)^42^

1 $${r}_{xy}=\frac{0.61\lambda }{NA}$$

being $\lambda$ the laser excitation wavelength and ${NA}$ the numerical aperture of the objective used. The shorter the wavelength, the higher is the lateral resolution (eq. (1)) as well as Raman scattering intensity as it is proportional to the $(\frac{1}{{\lambda }^{4}})$^43^. This is why short excitation wavelength and high laser power give the best signal-to-noise-ratio (S/N) as well as the best spatial resolution in Raman imaging. However, when measuring biological specimen the choice of the adequate Raman laser excitation wavelength depends greatly on the nature of the sample^44^. Biological samples often absorb in the UV/VIS range and can be changed or damaged if the applied beam intensity is too high. If the wavelength is very short and the exposure time too long, sample burning might occur due to absorption of UV radiation or an increase in the thermal energy^45,46^. Also lignin is known to be photodegraded by UV-Violet light, but no chemical degradation has been observed for wavelengths greater than 434 nm^47^. Auto-fluorescence of lignin is reported in the range of 515–600 nm^48^, while its absorption maximum lies in the UV region at about 350 nm, which causes the ultimate degradation of wood^49^, commonly observed by darkening in outdoor facades^50^. Yet for Raman imaging of plant cells and other biological materials, a submicron resolution is necessary to relate chemical changes to single cells or cell organelles or cell wall layers. Therefore, most routine Raman experiments are usually run with a VIS laser; *e.g*. with 532 nm laser a compromise between signal, resolution and non-destructive character is achieved.

Up to now, very few publications have dealt with changes of plant cell walls caused by laser exposure in the VIS range. Agarwal and Atalla (1986)^51^ reported the effects of the laser power on plant cell walls with excitation wavelength of 514.5 nm during Raman microprobe experiments. They stated that powers higher than 5 mW had effects on the intensity of the aromatic bands. Back then, the effect of the exposure time and kinetics of band ratios could not be studied as far too long acquisition times (58 min/spectrum) were necessary to record spectra with good S/N ratio. Interestingly, later on with a different wavelength (633 nm, 20 mW, in air) and still relatively high exposure time (15 s) Agarwal reported no changes in the main aromatic (lignin) band at 1600 cm^−1^ in the S_2_ cell wall layer with repetitive laser exposure^52^. In one study on thermomechanical pulps, laser-induced fluorescence was even useful to get information on quinone/hydroquinone groups, due to their molecular oxygen sensitivity when excited with 514.5 nm^53^.

Herein we study in detail the effect of 532 nm laser irradiation on lignified plant cell walls by repeated imaging experiments on different wood species and on the model plant *Arabidopsis thaliana*. We aim to elucidate the dependence of the spectral changes on laser power (expressed as laser intensity) and laser exposure time (expressed as energy density) and the implications on the structure of the lignin polymer. The observed spectral changes have been explained based on studies of lignin-model compounds under different environmental conditions. Controlled time series or repeated measurements can lead to new additional information on the aromatic nature of biological samples.

## Results and Discussion

### Repeated laser exposure of plant material changes its aromatic structure

**Figure 1:**
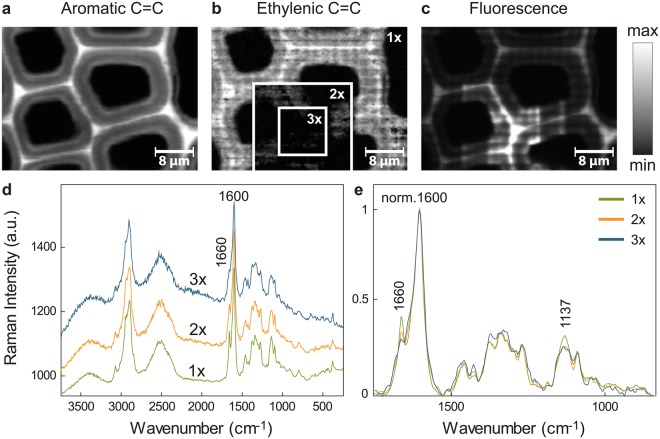
Repeated laser exposure causes chemical changes during Raman imaging of Spruce wood. (**a**) Integration over the main aromatic C=C stretching of lignin, (**b**) the ethylenic C=C stretching and the (**c**) fluorescent background. Integration over a specific Raman band gives a picture of the distribution and relative amount of the functional groups responsible for the band. The Raman images are scaled from the minimum (black) to the maximal intensity (white) of the integrated band in the image. The Raman scan shown is the last of three consecutive measurements of increasing size and thus comprises regions measured three times (3x), twice (2x) or just once (1x). The inserted frames depict the successive number of Raman images taken exemplarily in (**b**). The aromatic C=C remains stable over the three consecutive measurements (**a**). In contrast, the ethylenic C=C, attributed to coniferyl alcohol, decreases abruptly with increasing number of measurements (**b**). The fluorescence increases as shown by an increase of the background intensity at the area framed by “3x” (**b**). The common square (framed by “3x”) was used as a mask to average the spectra of the three consecutive Raman scans of exactly the same area for comparison (**d**). The spectra were normalized over the aromatic C=C ring stretching for visualization of changes in composition in (**e**).

Raman imaging was performed on a spruce microsection scanning an area with consecutive increasing size. The last and biggest measurement is shown in Fig. 1 and thus comprises regions measured three times (3x, inner square), two times (2x, frame around the inner square) and the outer part just once (1x). The laser power was set to the maximum (35 mW) for an exposure time of 0.13 s at every position (pixel). Figure 1a–c shows the distribution maps for the main aromatic band at 1600 cm^−1^ (C=C stretching of phenol ring), the C=C stretching of the ethenyl moiety of coniferyl alcohol at 1660 cm^−1^ and the fluorescence (by plotting changes in background intensity from 1800–2500 cm^−1^, where no Raman bands are visible), respectively. With increasing number of measurements, the overall intensity of the aromatic C=C at 1600 cm^−1^ remained relatively constant (Fig. 1a). However, the intensity of the ethylenic band at 1660 cm^−1^ decreased in the area comprising two and three measurements, as demonstrated by a darkening of these areas framed as “2x” and “3x” compared to the area marked as “1x” (Fig. 1b). Contrarily, the intensity in the fluorescence map increased at already irradiated positions (Fig. 1c). For comparison of the spectra, the smallest area measured (3x, corresponding to 900 spectra) was used as a frame for averaging exactly the same area of the first, second and third measurement (Fig. 1d). The most prominent changes were observed in the bands attributed to double bond stretches (doublet at 1600 and 1660 cm^−1^) and in the increasing intensity of the fluorescence background. For better visualization of the compositional changes, the spectra were normalized against the main aromatic band at 1600 cm^−1^ (Fig. 1e). This confirmed the decrease of the intensity of the C=C stretch at 1660 cm^−1^ with every measurement repetition. The band at approximately 1137 cm^−1^, attributed to coniferyl aldehyde^54^ experimented a broadening and a decrease in intensity. This behavior was proven to be a general phenomenon for lignified plant cell walls as it was observed in several tree and plant species, including pine (*Pinus nigra*), poplar (*Populus alba)* and *Arabidopsis thaliana* (see Supplementary Figs S1–S3, respectively).

### Energy density and influence of measurement repetition on the ethenyl/aromatic C=C ratio in wood

In the last section, laser power and integration time were kept constant. However, it is important to know if these parameters induce spectral changes above a certain threshold and/or measurement repetition.

**Figure 2:**
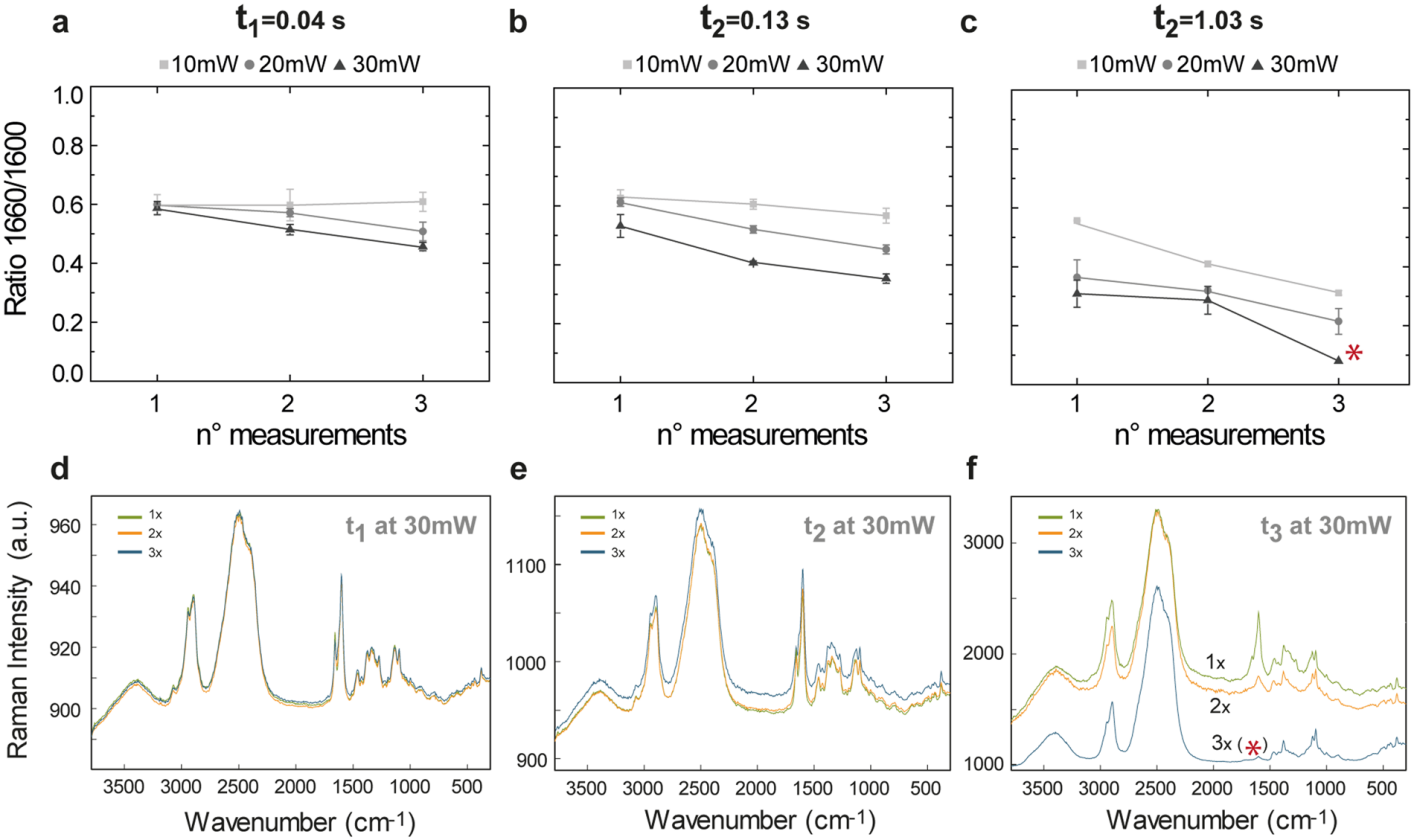
Change of the ethylenic/aromatic C=C ratio (1660/1600) during repeated Raman imaging of Spruce wood in dependence on laser power and exposure time. Ratio 1660/1600 vs number of measurements for different laser powers at three repetitive exposure times (**a**) t_1_, (**b**) t_2_ and (**c**) t_3_. With the smallest exposure time t_1_ the ratio shows a mild decrease for a laser power of 30 mW after the first measurement, but no changes are depicted for lower exposures. For an exposure time t_2_, the ratio 1660/1600 changed after the first measurement for laser powers higher or equal than 20 mW. With a laser power of 10 mW the ratio 1660/1600 only changed after the two consecutive measurements with an exposure time of 1.03 s. Example of the first, second and third measurement repetition for the highest laser power (30 mW) and exposure times t_1_, t_2_ and t_3_ are shown in (**d**–**f**), respectively.

The influence of the laser power and irradiation time on the intensities of the bands at 1600 and 1660 cm^−1^ of sequential measurements of spruce areas (including cell corner and cell wall) is shown in Fig. 2a–c. To monitor the changes, the ratio 1660/1600 was plotted. This compensates for differences in intensity of single bands due to changes of the focal plane. Clearly, the exposure time plays a major role in the final value of the 1660/1600 ratio, as it decreases with increasing exposure time and number of trial measurement. In addition, for constant exposure times, the band ratio decreased with increasing laser power (*i.e*. laser intensity). The most dramatic decrease was observed for the largest exposure time and highest number of measurement repetition (Fig. 2c). The measurements also showed that for the first two exposure times (t < 0.13 s), a power of 10 mW always delivers similar ratios independently of the number of sequential measurements. For the set up used, this threshold corresponds to an energy density of approximately 580 kJ/cm^2^.

The average spectra of the three consecutive images taken on the same area, at 30 mW and different exposure times (t_1_, t_2_ and t_3_), are displayed in Fig. 2d–f. For the extreme case (Fig. 2f), the ethylenic C=C band at 1660 cm^−1^ descended abruptly and after the third measurement no lignin bands (neither the 1600 cm^−1^ band, nor the 1660 cm^−1^ band) are present (marked with an asterisk in Fig. 2c) and only cellulose bands are visible in the spectrum. The fluorescence background also diminished after the third measurement.

Lignin is a phenolic compound polymerized *in vivo* from the so-called monolignols (p-coumaryl, coniferyl and sinapyl alcohol)^55^. The monolignols are synthesized in the cytosol^56^ and are deposited in a diffuse manner during secondary cell wall formation^57^. The ratio of the Raman bands 1660 and 1600 cm^−1^ has earlier been used to determine the polymerization degree of lignin since the ethenyl C=C groups are present in coniferyl alcohol and aldehyde, both precursors of lignin. Using a VIS laser with λ_ex_ = 633 nm, Morikawa *et al*.^58^ showed that after formation of the S3 cell wall layer in Japanese cypress the ratio did not change in cell corners located at positions with increasing distance from the cambium, since all lignin precursors are fully bound to the polymer. However, using a 532 nm laser, our results indicate that the amount of energy given to the sample during the measurements influences the value of the ratio 1660/1600, from the very first measurement, in areas at analogous positions *i.e*. same distance from cambium (see Fig. 2, measurement 1). In the same manner, by repeatedly measuring (up to three times) the same position, the ratio 1660/1600 in spruce also changed (Fig. 2, measurements 2 and 3), even at locations very far away from the cambial zone (approximately 7 cm) in the inner heartwood (data not shown). These observations highlight the importance of a careful selection of optimal measuring conditions, gathered in a single parameter, the energy density or fluence, during Raman imaging of plant material. The magnitude of the laser intensity (W/cm^2^) and energy density (kJ/cm^2^) is shown in Supplementary Fig. S4 for the optical objectives with magnifications of 20× (NA 0.4, air) and 100× (NA 1.4, oil), for the three different laser powers and irradiation times used in this work.

### Chemical changes in wood are related to changes in coniferyl alcohol

**Figure 3:**
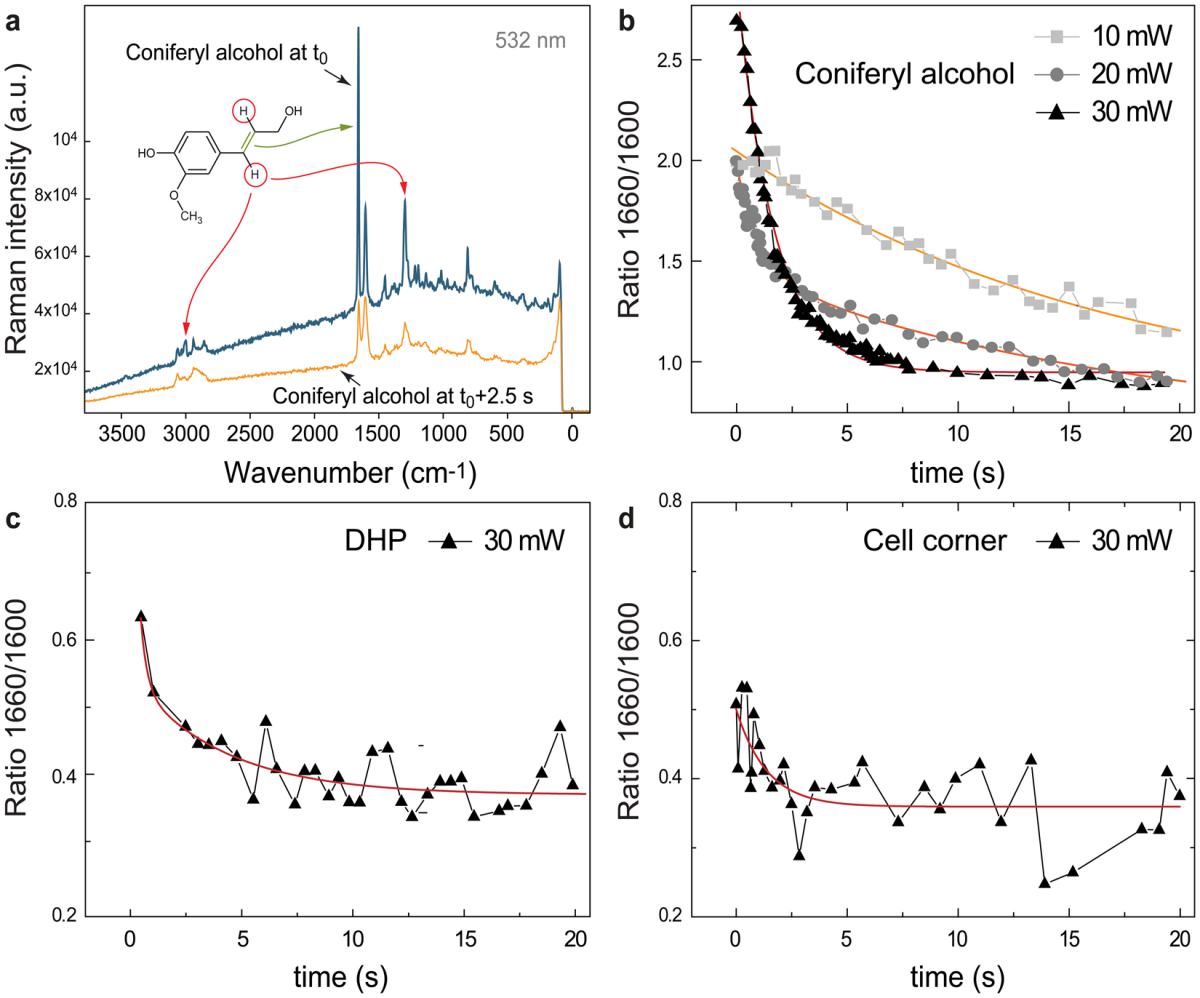
Spectral changes of coniferyl alcohol under laser exposure and time series of reference model compounds taken with λ_ex_ = 532 nm. (**a**) Raman spectra of coniferyl alcohol at t_0_, meaning measured at the earliest time possible, and at t_0_ + 2.5 s, with a laser power of 30 mW. The Raman bands affected are marked with an arrow depicting the functional groups that undergo a modification upon laser exposure in coniferyl alcohol. Most notably is the decrease of the C=C stretch at 1660 cm^−1^. Also two bands representing the C–H stretch (3013 cm^−1^) and bend (1297 cm^−1^) lose intensity. New bands around 2914 cm^−1^ (unsaturated C–H stretch) arise. Raman band intensities at 1600 and 1660 cm^−1^ measured over time from the three substances were used to calculate the ratio 1660/1600 displayed in (**b**) for coniferyl alcohol, for laser powers of 10, 20 and 30 mW, and (**c**) DHP and (**d**) cell corner of spruce wood, for a laser power of 30 mW. The ratio decay followed an exponential trend for all compounds. An exponential decay function was used to fit the ratio over time for all three compounds. For coniferyl alcohol, a stronger decay was observed the higher the laser power. The fitting parameters are displayed in Table 1.

The decrease in the ratio 1660/1600 in wood changed depending on the laser power and exposure time applied, as depicted in Fig. 2. The most affected band at 1660 cm^−1^ is attributed to C=O stretching of aldehydes and C=C stretching of the ethenyl moiety occurring in alcohol lignin monomers^59^ (monolignols), from which lignin is polymerized. To prove whether the aldehydes or alcohols are involved in the observed laser exposure changes, Raman spectra of them were acquired in time series experiments. With continuous laser irradiation (λ_ex_ = 532 nm) one Raman spectrum was collected every 0.04 s. Coniferyl and sinapyl aldehyde Raman spectra did not show any changes in the 1660 cm^−1^ band region over time (data not shown), whereas coniferyl and sinapyl alcohol did (Fig. 3a,b, shown for coniferyl alcohol.). Therefore, the changes were solely assigned to monolignols. In Fig. 3a the blue spectrum corresponds to the first measurement of coniferyl alcohol (zero time), whereas the orange spectrum was taken after 2.5 s. Similar as observed for wood (Fig. 2), the signal of C=C group decreased upon laser exposure. In addition, the CH stretching of C=C at 3015 cm^−1^ decreased and new bands arose in the region from 2990 to 2830 cm^−1^. These new bands, assigned to CH stretches of sp^3^-hybridized carbons likely come from the carbons of the former double bound of the 3-hydroxy-prop-1-en-1-yl moiety (named R_1_ in the remainder of the text) of coniferyl alcohol.

### Kinetics of the aromatic structural change in coniferyl alcohol, DHP (dehydrogenation polymer of lignin) and lignin

To compare the spectral changes of the monomer and the lignin polymer upon laser radiation, the time series procedure was followed for coniferyl alcohol, DHP (dehydrogenation polymer of lignin, an artificial synthetized lignin polymer), and a cell corner (CC) of Spruce, which is known to be composed of almost pure lignin. The ratio 1660/1600 was calculated from the band intensities and is depicted in Fig. 3b–d for coniferyl alcohol, DHP and the cell corner of spruce, respectively. The correspondent single spectra after irradiation of approximately 0.5 s and 20 s are shown in Supplementary Fig. S5a–c. The band intensities, from which the ratio was calculated, are depicted in Fig. S5d–f. For all laser powers, all compounds experienced an exponential decay in the band ratio over time. At 30 mW (laser intensity~1.4 × 10^6^ W/cm^2^), the quickest intensity decrease of the ratio was observed for coniferyl alcohol. By contrast, DHP and CC of Spruce showed a comparable slight decrease (Fig. 3b–d and Supplementary Fig. S5d–f).

In particular, for coniferyl alcohol, the laser power influences the rate of decrease of the band ratio (Fig. 3b): the higher the laser power, the faster the ratio decrease. For the same laser intensity, the initial ratios varied between coniferyl alcohol, DHP and CC of spruce, being the first the one with the highest ratio (~2.75). On the contrary, DHP and CC showed lower and similar ratios at the beginning (~0.5–0.65).

From the decay curve of the ratio 1660/1600 as a function of the exposure time, the rate of change ($k$) and half-life (${t}_{1/2}$) were evaluated for coniferyl alcohol, DHP and CC of spruce with the following exponential function:

2 $$y={A}_{1}\times {e}^{(\frac{-x}{\tau })}$$

Here $y$ is the ratio 1660/1600, $x$ is the irradiation time and $\tau$ is the characteristic mean life time which is $\tau \,=\,\frac{1}{k}\,=\,1.44\,\times \,{t}_{1/2}$, being $k$ the decay rate and ${t}_{1/2}$, the half-life time. The fitting results are shown in Table 1.

**Table 1:** Rate of change (k) and half life time (t_1/2_) of the ratio between the 1660/1600 (cm^−1^) Raman bands during time series for Coniferyl alcohol at 10, 20 and 30 mW, DHP at 30 mW and cell corner of Spruce microsection at 30 mW.

LPO (mW)	Coniferyl Alcohol			DHP	Cell corner Spruce
	10	20	30	30	30
τ (s)	16.88	17.14	1.67	2.75	1.39
		1.04			
k (s^−1^)	0.06	0.06	0.60	0.36	0.72
		0.96			
t_1/2_ (s)	11.70	11.88	1.16	1.91	0.96
		0.72			

Different laser powers were applied only to coniferyl alcohol, in order to determine the kinetics of the process. Due to the presence of noise when reducing the laser power, for DHP and cell corner, only the upper extreme (30 mW) was studied. During the time series measurement, the irradiation was continuous and one spectrum was taken every 0.04 s.

First, $k$ rose with increasing laser power for coniferyl alcohol, and in turn, ${t}_{1/2}$ diminished. At maximum power (30 mW), the value of the ratio changed at a rate of *k* = 0.06 s^−1^ and was half of the initial ratio after 1.16 s, which corresponded to an applied energy density of about 1600 kJ/cm^2^. Interestingly, with 20 mW (laser intensity~9.2 × 10^5^W/cm^2^) two processes are involved (double exponential decay). A quick process is observed at short times (*k* = 0.96 s^−1^) while for longer times, a slow process dominates. The slowest ratio change was recorded for 10 mW (*k* = 0.06 s^−1^) (laser intensity~4.6 × 10^5^W/cm^2^), similar to the second process when applying 20 mW.

When comparing the coniferyl alcohol with model compounds taken at the maximum laser power (30 mW) (Fig. 3b,c, Table 1), DHP presented the lowest $k$ and highest ${t}_{1/2}$. DHP is an artificial compound polymerized to a very high degree, and almost no free coniferyl alcohol should be present. In the case of CC, the trend line also depicted an exponential trend and the kinetic parameters are comparable with the values obtained for coniferyl alcohol for the same laser power. This might be an indication of the presence of coniferyl alcohol molecules in cell corners of native wood. It could be hypothesized that this is a further polymerization (in german more appropriately described by the word “Nachpolymerisierung”) of lignin. Also the fact that polymerization studies on lignin showed an exponential decay of the number of lignin guaiacyl monomer units over time^60^ points to this process.

### VIS induced changes of coniferyl alcohol: plausible mechanisms

**Figure 4:**
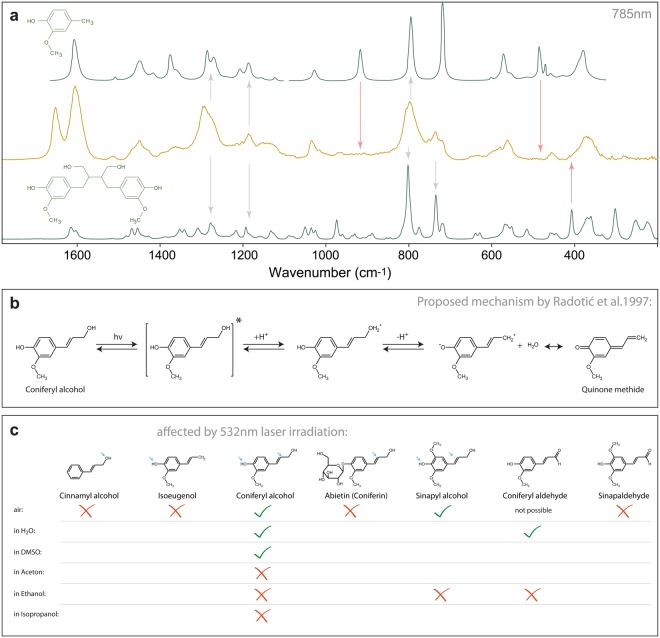
Raman spectra of coniferyl alcohol and its degradation and polymerization product, proposed reaction mechanism and summary of the references measured. (**a**) Comparison of the laser-exposed spectrum of coniferyl alcohol with two reference substances miming its degradation (top) and a polymerization product (bottom). Neither of the two compounds fits perfectly (red arrows), but are sufficiently similar to deduce that the aromatic ring is still present (grey arrows, also the aromatic stretch at 1600 cm^−1^ is still here). (**b**) The reaction mechanism proposed by Radotic *et al*.^65^. (**c**) Molecular structure of the five compounds measured over time. The references present the same backbone, but differ in the OH configuration. Note that only those compounds, which bear both an OH-group at the ring and at the propenyl moiety, were affected by the 532 nm laser.

Lignin in wood is known to be degraded by UV light^49^. However, longer wavelengths *i.e*. blue light, have been shown to not cause any changes in the fluorescence emission spectra of lignin after repeated measurement^61^. Yet the present work shows that green light induces chemical changes in wood and lignin reference model compounds. From the groups affected in the Raman spectra, the changes have been attributed to monolignols. The drop in the ratio 1660/1600 indicated a decrease in the relative number of ethenyl (C=C) groups, which might occur when these groups are degraded or when alcohol monomers polymerize into lignin, as occurs in nature^58,62^. Therefore in a next step the spectrum of coniferyl alcohol after laser exposure was compared with the spectra of the two possible outcomes, a phenolic ring without R_1_ (degradation product), and a dimer of coniferyl alcohol (polymerization product) (Fig. 4a). Although in theory the reduction of the ethenyl C=C could be explained by both mechanisms, neither the polymer nor the degraded component spectra coincide completely with the laser induced product of coniferyl alcohol.

4-Vinylphenols like coniferyl alcohol can undergo photochemical reactions upon UV irradiation^63^ and the polymerization of coniferyl alcohol was already studied in several papers by Radotic and co-workers^64,65^. They produced so-called photopolymers by exposing coniferyl alcohol to UV light. Interestingly, they did not find radicals to be involved in the reaction^65^, which is in contrast to other research on 4-vinylphenols, where radical-reactions were assumed^66,67^. They therefore proposed an alternative reaction pathway involving protons, which is shown in Fig. 4b. This reaction involves two OH-groups, one attached to the ring, the other one attached to the ethenyl moiety. Therefore, additional Raman time series of similar substances with varying OH-configuration were recorded under equal experimental conditions. The data revealed that 532 nm irradiation only had an effect when both ring and propenyl moiety bear an OH-group (Fig. 4c). This is an indication that only monolignols can be responsible for the observed laser induced changes in plant cell walls.

Another fact described by Radotic *et al*.^65^ was that coniferyl alcohol shows an absorption band at 350 nm under UV light exposure, which they tentatively assigned to quinone methide, a strong fluorescence compound. This band was suppressed completely when they recorded absorption spectra of coniferyl alcohol in ethanol, pentanole and DMSO (dimethyl sulfoxide). Further experiments of us revealed that the laser degradation of the 532 nm-susceptible substances *i.e*. coniferyl and sinapyl alcohol did not happen in acetone, ethanol or isopropanol as solvent, but changed in water, DMSO and air (in the latter air moisture might be responsible) (Fig. 4c). These facts support the proposed reaction by Radotic *et al*.^65^, that quinone methide is involved in the reaction and that the process can be controlled over this species. In contrast to sinapyl and coniferyl alcohol, Raman measurements of DHP and spruce wood in EtOH suffered chemical changes after repeated measurement. The decrease of the 1660 cm^−1^ band was accompanied by the rise of a band at 1630 cm^−1^ (data not shown). This band could derive from either conjugated C=O or C=C stretches^68^ or represent an aromatic ring stretch. Varsanyi^69^ gives a spectral range up to 1628 cm^−1^ already for 1,4-dilight substituted rings (*i.e*. H-units of lignin), and assigns values up to 1660 cm^−1^ when more substituents are present. Due to the inherent complexity of the lignin polymer, this band has to be left aside for now, as we cannot relate it to any of the processes described above.

At 532 nm, coniferyl and sinapyl alcohol showed no molecular absorption band (see Supplementary Fig. S6), which rules out polymerization mechanisms running over an excited state. Since these compounds do not absorb at the wavelength used, a photoinduced electron-transfer mechanism can be excluded. The results showed that the energy transferred to the molecules was not sufficient to dissociate bonds^70^, but may be enough to oxidize them. Taking into account the oxidation energy of coniferyl alcohol in aqueous solution, 1.03 × 10^22^ kJ^71^, and the energy density applied, the theoretical number of coniferyl alcohol molecules that could be “activated” was calculated (Table 2). Only with the initial energy density given by the minimum laser power (Supplementary Fig. S4), around 1.96 × 10^23^ molecules of coniferyl alcohol could be theoretically oxidized (Table 2). This number increased linearly with time due to the continuous irradiation given when performing the time series experiments. This agrees with a previous study on diethyl ether clusters, were a 532 nm laser was used for the ionization^72^.

**Table 2:** Maximum theoretical initial number of molecules of coniferyl alcohol able to oxidize monolignols for different energy density at t_1_ = 0.04 s.

LPO (mW) (λ_ex_ = 532 nm)	Integration time (s)	Max. n° MOL/cm^2^ (CA)	
		20 × NA = 0. 4	100x inmersion oil NA = 1.4
10	t_1_ = 0.04	1.96 × 10^23^	1.84 × 10^24^
20	t_1_ = 0.04	3.92 × 10^23^	3.69 × 10^24^
30	t_1_ = 0.04	5.88 × 10^23^	5.53 × 10^24^

The ionization energy of coniferyl alcohol is about 0.64 eV/molecule^71^ which corresponds to 1.22 × 10^−21^ kJ/molecule.

When the measurements of coniferyl alcohol and wood were carried out at λ_ex_ = 785 nm, no changes in the ratio 1660/1600 were observed over time, even after 200 s at 220 mW, neither for native wood, nor coniferyl and sinapyl alcohol (data not shown). The energy applied corresponded to energies comparable to lower irradiation times and power with an λ_ex_ = 532 nm. This might imply that the minimum photon energy plays a role in the oxidation of coniferyl alcohol. The energy carried per photon is described as

3 $${\rm{{\rm E}}}=h\times \,\frac{c}{\lambda }$$

where $h$ is the Planck constant, $c$ the speed of light and $\lambda$ the wavelength of the excitation source. The energy was about 3.74 × 10^−22^ kJ for a λ_ex_ = 532 nm while for λ_ex_ = 785 nm the energy was reduced to 2.53 × 10^−22^ kJ. Although both energies would be theoretically enough to “activate” coniferyl alcohol^71^, only the shorter wavelength was successful.

Time series of coniferyl and sinapyl aldehyde did not depict any changes of the band ratio over time. Neither did reference substances bearing only one OH group, which proved that monolignols are the ones affected by 532 nm laser irradiation. Beside coniferyl and sinapyl alcohol, also DHP and spruce wood experienced a decrease of the number of ethylenic C=C groups, which indicates the presence of coniferyl alcohol molecules in DHP (about 20% as estimated by H-NMR) and wood. Adding EtOH, isopropanol or acetone suppressed the modification of monolignols, which has been an indication that a polymerization process takes place. For a degradation mechanism, the same result would be expected for all solvents. Nevertheless, adding the same solvents to DHP and native wood caused changes in the band ratio accompanied by a new band, which due to the heterogeneity of the native lignin could not be explained. For Raman imaging of spruce wood, when using the highest exposure time (t_3_) and the highest laser power (30 mW), the lignin band disappeared after the third consecutive measurement (see Fig. 2c,d, marked with asterisk). It seems that first, the energy is dissipated in further polymerizing the unbound monolignols into lignin. When the energy is too high, the polymerization is fully completed and the polymer overheats and finally degrades. From the results obtained in this work, we can state that the 532 nm laser irradiation only affects coniferyl/sinapyl alcohol units, because only these molecules have two OH-groups necessary for the induced photo-polymerization. According to this work, coniferyl/sinapyl alcohol units that exist in DHP and wood can be probed selectively by times series experiments.

## Experimental

### Sample preparation and sectioning

4-year-old spruce trees (*Picea abies* (L.) Karst.) were obtained from the company Lieco (Austria). Sections of 5 µm were obtained by cryomicrotomy (CM3050 S, Leica, Austria). Pine (*Pinus sylvestris L*.), Arabidopsis (*Arabidopsis thaliana*) and Poplar (*Populus alba L*.) material was obtained from different areas and was sectioned (3 to 15 µm thick) by rotary microtomy (RM2255, Leica, Austria) at room temperature in the presence of deuterium oxide (D_2_O) to maintain the native moisture. The sections were placed on a glass slide with a drop of D_2_O and were covered with a glass coverslip (No. 1.5, 0.17 mm thick). The coverslip was sealed with nail polish to avoid D_2_O evaporation during the measurements.

Abietin (90% purity), Coniferyl alcohol (98%), Coniferyl aldehyde (98%), Isoeugenol (98%) and Sinapinaldehyde (98%) were purchased from Sigma Aldrich (Austria) and used as received.

Sinapyl and cinnamyl alcohol were made by NaBH_4_ reduction from their respective aldehydes, which were purchased from Sigma Aldrich (Austria) and used as received. Details can be found in the Supplementary Methods S7.

Dehydrogenation polymer of ferulic acid was obtained from Grigory Zinovyev, from the Division of Chemistry and Renewable Resources (Department of Chemistry, University of Natural Resources and Life Sciences, Vienna, Austria).

### Confocal Raman Microscopy

Raman images of all samples were taken with a Raman microscope (Alpha300RA, WITec GmbH, Germany). The samples were exited with a linear polarized (0°) coherent compass sapphire VIS laser with λ_ex_ = 532 nm (WITec GmbH, Germany) through a 100x oil immersion objective (numerical aperture (NA) = 1.4, coverslip correction 0.17 mm) (Carl Zeiss, Germany). The scattered Raman signal was collected through the same objective and directed through an optic multifiber (50 µm diameter) to a spectrometer (UHTS 300 WITec, Germany) (600 g.mm^−1^ grating, spectral resolution about 3.8 cm^−1^, maximum and minimum error of about 4.8 and 2.9 cm^−1^, respectively) and finally to the CCD camera (Andor DU401 BV, Belfast, North Ireland). The maximum spatial resolution that can be achieved is given by r = 0.61λ/NA, which for the parameters and confocal set up given is about 230 nm. The Control Four (WITec GmbH, Germany) acquisition software was used for the Raman imaging set up.

Three concentric images of increasing size were taken on the samples in order to illustrate the effect of the repeated laser irradiation on the intensity of specific aromatic bands during the measurements. For this example, the laser power was set to a maximum of 35 mW, and the integration time to 0.13 s, which corresponded to a laser intensity of 1.3 × 10^8^ W/cm^2^ on the sample.

The laser power and the integration time (exposure time) were also changed in order to study the effect of the excitation energy on the aromatic bands. In order to record this effect, 3 sequential images of equal size were measured at the same position. For each set up, 3 replicates were measured for statistical analysis. The replicates were obtained at areas including cell wall and cell corner, located at the same distance from the outer part of the stem (about 50 µm from the cambial cells), which ensured a similar initial chemical profile. This procedure was carried out for three different laser powers (10, 20, and 30 mW, power measured before the objective) and exposure times (t_1_ = 0.04 s, t_2_ = 0.13 s, t_3_ = 1.03 s). The laser intensities and energy densities for each set up can be found in Supplementary Material S4.

The changes in the Raman spectrum of coniferyl and sinapyl alcohol, coniferyl aldehyde, sinapinaldehyde, cinnamyl alcohol, isoeugenol and abietin were monitored by performing time series experiments with an accumulative exposure time of 0.04 s and laser powers (at λ_ex_ = 532 nm) of 10, 20 and 30 mW, respectively. In this case, the compounds were measured dry and with a 20x objective (NA 0.4) without the presence of a coverslip. In order to compare the changes during sample excitation, the same procedure was applied for DHP and a cell corner of a Spruce microsection. Furthermore, coniferyl alcohol was measured in ethanol, acetone, isopropanol (UV/IR grade, Carl Roth, Germany), DMSO (99.5%, Sigma Aldrich, Austria) and water (Merck Millipore) under a coverslip on an objective slide. Sinapyl alcohol was only measured air-dried and in ethanol.

Coniferyl and sinapyl alcohol were also measured over time using a near infrared laser with λ_ex_ = 785 nm. 785 nm experiments were conducted on the same instrument, using a linear polarized XTRA II laser (Toptica Photonics, Germany) The scattering was detected with an optic multifiber (100 µm) directed to a spectrometer (UHTS 300, WITec, Germany) equipped with blazed gratings (600 and 1200 g.mm^−1^, BLZ 750 nm) and a CCD camera (Andor DU401 DD, Belfast, North Ireland).

### Fluence

The fluence or energy density of a laser refers to the total amount of energy per unit of area. The fluence on the sample changes depending on the laser power set, the irradiation time and the objective used, *i.e*. transmittance and numerical aperture (NA), since these parameters determine as well the laser focus spot. In order to determine the energy density (kJ/cm^2^) on the sample (see Supplementary material S4) the energy of the laser and the illuminated area were determined. First, the laser power before the objective was measured with a power meter for the specific excitation wavelength (PM100D, Thorlabs, USA). The laser power after the objective was corrected for the transmittance factor of the objective and multiplied by the exposure time (t_1_, t_2_ or t_3_), since the energy density is a function of time, in order to obtain the energy irradiated on the sample. In addition, the maximal lateral resolution (eq. (1)) was used as radius for calculating the laser spot area, given as a circle with a Gaussian distribution.

With the purpose of confirming the possibility of an ionization of the hydroxyl group as a starting mechanism for the polymerization of coniferyl alcohol when VIS radiation was applied, the maximum theoretical number of OH-groups of the molecule coniferyl alcohol that could be oxidized with the applied energy per unit of area was calculated (see Table 2).

#### Vibrational analysis

The equilibrium geometry and vibrational analysis of the molecules were calculated using the GAMESS package^73,74^. The SCF-DFT functional B3LYP together with the 6–311G basis set was used for all calculations. Vibrational modes were displayed with MacMolPlt v7.7^75^ The bands were assigned according to our (polarization) measurements, our calculations as well as to standard literature^68,69,76^ and to studies of related compounds^77–80^

### Data analysis

For visualization of the effect, every first, second and third measurement (image) of each replicate was averaged. The spectra are shown without pre-processing and after baseline correction and normalization against the 1600 cm^−1^ band.

For the quantification of the effect of the exposure time and laser power on the 1600 and 1660 cm^−1^ Raman bands, the average spectrum of each image was calculated in the software Project Four Plus 4.1 (WITec GmbH, Germany). The spectra were extracted and the Rayleigh line cut before the average spectra were baseline corrected (Rubber band mode, 10 iterations, and 64 points) in OPUS (Bruker, Germany). After baseline correction, the height of the bands at 1600 and 1660 cm^−1^ was determined. The band height was set as the intensity from a virtual baseline, fitted to the right and left lowest intensities of the band of interest, to the maximum of the band. The ratio between the two band heights 1660/1600 was calculated for each average spectrum and the population standard deviation between replicates determined.

From the time series on coniferyl alcohol, first, the spectra were extracted, the Rayleigh line removed and baseline corrected (Rubber band mode, 10 iterations, 64 points) in OPUS (Bruker, Germany). The band heights were read with the same method for spruce images, DHP and cell corner of Spruce.

## Conclusions

Studying aromatic components in biological materials by Raman imaging using the 532 nm laser irradiation has to include careful adjustment of experimental parameters, namely laser power and irradiation time. In lignified plant cell walls as well as in an artificial lignin polymer (DHP) a clear change in the ethenyl/aromatic C=C ratio was observed at high energy densities. Studies on references components and under different environmental conditions (solvents) could explain the effect as a polymerization/degradation of monolignols. Time series experiments are recommended to adjust experiment parameters and derive additional information on monolignols located within the secondary plant cell wall and the junctions between cells.

## Electronic supplementary material

Supplementary Material and Methods

## References

[1] Mandal SM, Chakraborty D, Dey S (2010). Phenolic acids act as signaling molecules in plant-microbe symbioses. Plant Signaling & Behavior.

[2] Nicholson RL, Hammerschmidt R (1992). Phenolic-Compounds and Their Role in Disease Resistance. Annu Rev Phytopathol.

[3] Pieterse CMJ, van Loon LC (1999). Salicylic acid-independent plant defence pathways. Trends in Plant Science.

[4] Ramamurthy MS, Maiti B, Thomas P, Nair PM (1992). High-Performance Liquid-Chromatography Determination of Phenolic-Acids in Potato-Tubers (Solanum-Tuberosum) during Wound-Healing. J Agr Food Chem.

[5] Gindl W (2002). Comparing Mechanical Properties of Normal and Compression Wood in Norway Spruce: The Role of Lignin in Compression Parallel to the Grain. Holzforschung.

[6] Gindl W, Teischinger A (2002). Axial compression strength of Norway spruce related to structural variability and lignin content. Composites Part A: Applied Science and Manufacturing.

[7] Laschimke R (1989). Investigation of the wetting behaviour of natural lignin - a contribution to the cohesion theory of water transport in plants. Thermochimica Acta.

[8] Voelker SL, Lachenbruch B, Meinzer FC, Kitin P, Strauss SH (2011). Transgenic poplars with reduced lignin show impaired xylem conductivity, growth efficiency and survival. Plant Cell Environ.

[9] Boerjan W, Ralph J, Baucher M (2003). Lignin biosynthesis. Annu Rev Plant Biol.

[10] Monties B (1991). Plant-Cell Walls as Fibrous Lignocellulosic Composites - Relations with Lignin Structure and Function. Anim Feed Sci Tech.

[11] Balakshin M, Capanema E, Gracz H (2011). Chang, H.-m. & Jameel, H. Quantification of lignin–carbohydrate linkages with high-resolution NMR spectroscopy. Planta.

[12] Parthasarathi R, Romero RA, Redondo A, Gnanakaran S (2011). Theoretical Study of the Remarkably Diverse Linkages in Lignin. The Journal of Physical Chemistry Letters.

[13] Lora JH, Glasser WG (2002). Recent industrial applications of lignin: a sustainable alternative to nonrenewable materials. Journal of Polymers and the Environment.

[14] Zakzeski J, Bruijnincx PC, Jongerius AL, Weckhuysen BM (2010). The catalytic valorization of lignin for the production of renewable chemicals. Chemical reviews.

[15] Sluiter A (2008). Determination of structural carbohydrates and lignin in biomass. Laboratory analytical procedure.

[16] Chen F, Dixon RA (2007). Lignin modification improves fermentable sugar yields for biofuel production. Nature biotechnology.

[17] Baucher M, Halpin C, Petit-Conil M, Boerjan W (2003). Lignin: Genetic Engineering and Impact on Pulping. Critical Reviews in Biochemistry and Molecular Biology.

[18] Ralph J (2006). Effects of coumarate 3-hydroxylase down-regulation on lignin structure. J Biol Chem.

[19] Ehlting J (2005). Global transcript profiling of primary stems from Arabidopsis thaliana identifies candidate genes for missing links in lignin biosynthesis and transcriptional regulators of fiber differentiation. Plant J.

[20] Schuetz M, Douglas C, Samuels L, Ellis B (2014). Manipulating lignin deposition. Can J Plant Sci.

[21] Ragauskas, A. J. *et al*. Lignin Valorization: Improving Lignin Processing in the Biorefinery. *Science* **344**10.1126/science.1246843 (2014). 10.1126/science.1246843 24833396

[22] Donaldson LA, Singh AP, Yoshinaga A, Takabe K (1999). Lignin distribution in mild compression wood of Pinus radiata. Can J Bot.

[23] Koch G, Kleist G (2001). Application of scanning UV microspectrophotometry to localise lignins and phenolic extractives in plant cell walls. Holzforschung.

[24] Sander C, Koch G (2001). Effects of acetylation and hydrothermal treatment on lignin as revealed by cellular UV-spectroscopy in Norway spruce (Picea abies [L.] Karst.). Holzforschung.

[25] Scott JAN, Procter AR, Fergus BJ, Goring DAI (1969). Application of Ultraviolet Microscopy to Distribution of Lignin in Wood - Description and Validity of Technique. Wood Sci Technol.

[26] Butler HJ (2016). Using Raman spectroscopy to characterize biological materials. Nat Protoc.

[27] Brauchle, E., Thude, S., Brucker, S. Y. & Schenke-Layland, K. Cell death stages in single apoptotic and necrotic cells monitored by Raman microspectroscopy. *Sci Rep-Uk* **4**10.1038/srep04698 (2014). 10.1038/srep04698 PMC3986703 24732136

[28] Michael, R. *et al*. Hyperspectral Raman imaging of neuritic plaques and neurofibrillary tangles in brain tissue from Alzheimer’s disease patients. *Sci Rep-Uk* **7**10.1038/s41598-017-16002-3 (2017). 10.1038/s41598-017-16002-3 PMC5688091 29142266

[29] Prats-Mateu B (2017). Label‐free live cell imaging by Confocal Raman Microscopy identifies CHO host and producer cell lines. Biotechnology Journal.

[30] Piredda P, Berning M, Boukamp P, Volkmer A (2015). Subcellular Raman microspectroscopy imaging of nucleic acids and tryptophan for distinction of normal human skin cells and tumorigenic keratinocytes. Analytical chemistry.

[31] Mandair, G. S. & Morris, M. D. Contributions of Raman spectroscopy to the understanding of bone strength. *BoneKEy Rep* **4**10.1038/bonekey.2014.115 (2015). 10.1038/bonekey.2014.115 PMC4296861 25628882

[32] Paschalis EP (2017). Fragility Fracture Incidence in Chronic Obstructive Pulmonary Disease (COPD) Patients Associates With Nanoporosity, Mineral/Matrix Ratio, and Pyridinoline Content at Actively Bone-Forming Trabecular Surfaces. J Bone Miner Res.

[33] Wang, H. Q. *et al*. *In vivo* coherent Raman imaging of the melanomagenesis-associated pigment pheomelanin. *Sci Rep-Uk* **6**10.1038/srep37986 (2016). 10.1038/srep37986 PMC5125099 27892516

[34] Gierlinger N, Schwanninger M (2006). Chemical imaging of poplar wood cell walls by confocal Raman microscopy. Plant Physiol.

[35] Agarwal UP (2006). Raman imaging to investigate ultrastructure and composition of plant cell walls: distribution of lignin and cellulose in black spruce wood (Picea mariana). Planta.

[36] Altangerel N (2017). *In vivo* diagnostics of early abiotic plant stress response via Raman spectroscopy. Proceedings of the National Academy of Sciences.

[37] Prats-Mateu, B., Hauser, M.-T., Heredia, A. & Gierlinger, N. Waterproofing in Arabidopsis: Following phenolics and lipids *in situ* by Confocal Raman Microscopy. *Frontiers in Chemistr*y **4**10.3389/fchem.2016.00010 (2016). 10.3389/fchem.2016.00010 PMC4770935 26973831

[38] Agarwal, U. P. & Ralph, S. A. Quantitation of lignin in grasses by near-IR FT-Raman spectroscopy. *Abstr Pap Am Chem S* **243** (2012).

[39] Agarwal UP, McSweeny JD, Ralph SA (2011). FT-Raman Investigation of Milled-Wood Lignins: Softwood, Hardwood, and Chemically Modified Black Spruce Lignins. J Wood Chem Technol.

[40] Agarwal UP, Obst JR, Cannon AB (1996). Lignin analysis by FT-Raman spectroscopy. Abstr Pap Am Chem S.

[41] Barsberg S, Matousek P, Towrie M (2005). Structural Analysis of Lignin by Resonance Raman Spectroscopy. Macromolecular Bioscience.

[42] Paddock, S. W. & Eliceiri, K. W. In *Confocal Microscopy: Methods and Protocols* (ed. Stephen W. Paddock) 9–47 (Springer New York, 2014).

[43] Milonni, P. W. & Eberly, J. H. In *Laser Physics* 331–400 (John Wiley & Sons, Inc., 2010).

[44] Kerr LT, Byrne HJ, Hennelly BM (2015). Optimal choice of sample substrate and laser wavelength for Raman spectroscopic analysis of biological specimen. Anal Methods-Uk.

[45] Jirasek A (2006). Discrimination between UV radiation‐induced and thermally induced spectral changes in AT‐paired DNA oligomers using UV resonance Raman spectroscopy. Journal of Raman Spectroscopy.

[46] LaPlant, F. Lasers, spectrographs, and detectors. *Emerging Raman Applications and Techniques in Biomedical and Pharmaceutical Fields* 1–24 (2010).

[47] Kataoka Y, Kiguchi M, Williams RS, Evans PD (2007). Violet light causes photodegradation of wood beyond the zone affected by ultraviolet radiation. Holzforschung.

[48] Donaldson LA, Radotic K (2013). Fluorescence lifetime imaging of lignin autofluorescence in normal and compression wood. J Microsc-Oxford.

[49] Cogulet A, Blanchet P, Landry V (2016). Wood degradation under UV irradiation: A lignin characterization. J Photoch Photobio B.

[50] Laskowska A, Dobrowolska E, Boruszewski P (2016). The impact of ultraviolet radiation on the colour and wettability of wood used for facades. Drewno.

[51] Agarwal UP, Atalla RH (1986). *In-situ* Raman microprobe studies of plant cell walls: Macromolecular organization and compositional variability in the secondary wall of Picea mariana (Mill.) B.S.P. Planta.

[52] Agarwal UP (2006). Raman imaging to investigate ultrastructure and composition of plant cell walls: distribution of lignin and cellulose in black spruce wood (Picea mariana). Planta.

[53] Agarwal UP (1998). Assignment of the Photoyellowing-Related 1675 cm^−1^ Raman/IR Band to P-Quinones and Its Implications to the Mechanism of Color Reversion in Mechanical Pulps. J Wood Chem Technol.

[54] Agarwal, U. P. An overview of Raman spectroscopy as applied to lignocellulosic materials. *Advances in lignocellulosics characterization* 201-225 (1999).

[55] Donaldson LA (2001). Lignification and lignin topochemistry - an ultrastructural view. Phytochemistry.

[56] Vanholme R, Demedts B, Morreel K, Ralph J, Boerjan W (2010). Lignin Biosynthesis and Structure. Plant Physiol.

[57] Schuetz M (2014). Laccases direct lignification in the discrete secondary cell wall domains of protoxylem. Plant Physiol.

[58] Morikawa Y, Yoshinaga A, Kamitakahara H, Wada M, Takabe K (2010). Cellular distribution of coniferin in differentiating xylem of Chamaecyparis obtusa as revealed by Raman microscopy. Holzforschung.

[59] Agarwal UP, Ralph SA (2008). Determination of ethylenic residues in wood and TMP of spruce by FT-Raman spectroscopy. Holzforschung.

[60] van Parijs FRD, Morreel K, Ralph J, Boerjan W, Merks RMH (2010). Modeling Lignin Polymerization. I. Simulation Model of Dehydrogenation Polymers. Plant Physiol.

[61] Donaldson L, Radotić K, Kalauzi A, Djikanović D, Jeremić M (2010). Quantification of compression wood severity in tracheids of Pinus radiata D. Don using confocal fluorescence imaging and spectral deconvolution. Journal of Structural Biology.

[62] Hanninen T, Kontturi E, Vuorinen T (2011). Distribution of lignin and its coniferyl alcohol and coniferyl aldehyde groups in Picea abies and Pinus sylvestris as observed by Raman imaging. Phytochemistry.

[63] Uppalapati S, Chada S, Engelhard MH, Yan M (2010). Photochemical Reactions of Poly (4‐vinylphenol) Thin Films. Macromolecular Chemistry and Physics.

[64] Radotic K, Todorovic S, Zakrzewska J, Jeremic M (1998). Study of photochemical reactions of coniferyl alcohol. II. Comparative structural study of a photochemical and enzymatic polymer of coniferyl alcohol. Photochem Photobiol.

[65] Radotic K, Zakrzewska J, Sladic D, Jeremic M (1997). Study of photochemical reactions of coniferyl alcohol I. Mechanism and intermediate products of UV radiation-induced polymerization of coniferyl alcohol. Photochem Photobiol.

[66] Rodriguez-Evora Y, Schepp NP (2005). Reactivity of 4-vinylphenol radical cations in solution: implications for the biosynthesis of lignans. Organic & biomolecular chemistry.

[67] DellaGreca M (2008). Lignans by photo-oxidation of propenyl phenols. Photoch Photobio Sci.

[68] Colthup, N. B., Daly, L. H. & Wiberley, S. E. *Introduction to Infrared and Raman Spectroscopy*. 3rd edn, (Academic Press, 1990).

[69] Varsányi, G. *Vibrational Spectra of Benzene Derivatives*. (Academic Press, 1969).

[70] Blanksby SJ, Ellison GB (2003). Bond dissociation energies of organic molecules. Accounts Chem Res.

[71] Wei K, Luo S-W, Fu Y, Liu L, Guo Q-X (2004). A theoretical study on bond dissociation energies and oxidation potentials of monolignols. Journal of Molecular Structure: Theochem.

[72] Zhang NZ, Wang WG, Zhao WD, Han FL, Li HY (2010). Multiply ionization of diethyl ether clusters by 532 nm nanosecond laser: The influence of laser intensity and the electron energy distribution. Chem Phys.

[73] Schmidt MW (1993). General Atomic and Molecular Electronic Structure System. Comput. Chem..

[74] Gordon, M. S. & Schmidt, M. W. In *Theory and Applications of Computational Chemistry: the first forty years* (eds C. E. Dykstra, G. Frenking, K. S. Kim, & G. E. Scuseria) 19 (Elsevier, 2005).

[75] Bode, B. M. & Gordon, M. S. *J. Mol. Graphics Mod*. **16** 6 (1998). 10.1016/s1093-3263(99)00002-9 10434252

[76] Mayo, D. W., Miller, F. A. & Hannah, R. W. *Course Notes on the Interpretation of Infrared and Raman Spectra*. (John Wiley & Sons, Inc, 2003).

[77] Larsen KL, Barsberg S (2010). Theoretical and Raman Spectroscopic Studies of Phenolic Lignin Model Monomers. J. Phys. Chem. B.

[78] Chowdhry BZ, Ryall JP, Dines TJ, Mendham AP (2015). Infrared and Raman Spectroscopy of Eugenol, Isoeugenol, and Methyl Eugenol: Conformational Analysis and Vibrational Assignments from Density Functional Theory Calculations of the Anharmonic Fundamentals. The journal of physical chemistry. A.

[79] Badawi HM, Förner W (2011). Vibrational spectra and assignments of 3-phenylprop-2-en-1-ol (cinnamyl alcohol) and 3-phenyl-1-propanol. Journal of Molecular Structure.

[80] Sebastian S, Sundaraganesan N, Manoharan S (2009). Molecular structure, spectroscopic studies and first-order molecular hyperpolarizabilities of ferulic acid by density functional study. Spectrochim Acta A.

