# Supplementary material for: Following laser induced changes of plant phenylpropanoids by Raman microscopy
Source: Sci Rep. 2018 Aug 7;8:11804. doi: 10.1038/s41598-018-30096-3 (PMC6081397; doi:10.1038/s41598-018-30096-3)
Supplement: Supplementary file 1 — Supplementary Material and Methods [file 41598_2018_30096_MOESM1_ESM.docx]

**Following laser induced changes of plant phenylpropanoids by Raman Microscopy**

Batirtze Prats-Mateu, Peter Bock, Martina Schroffenegger, José Luis Toca-Herrera and Notburga Gierlinger

**Supplementary Figures and Tables**


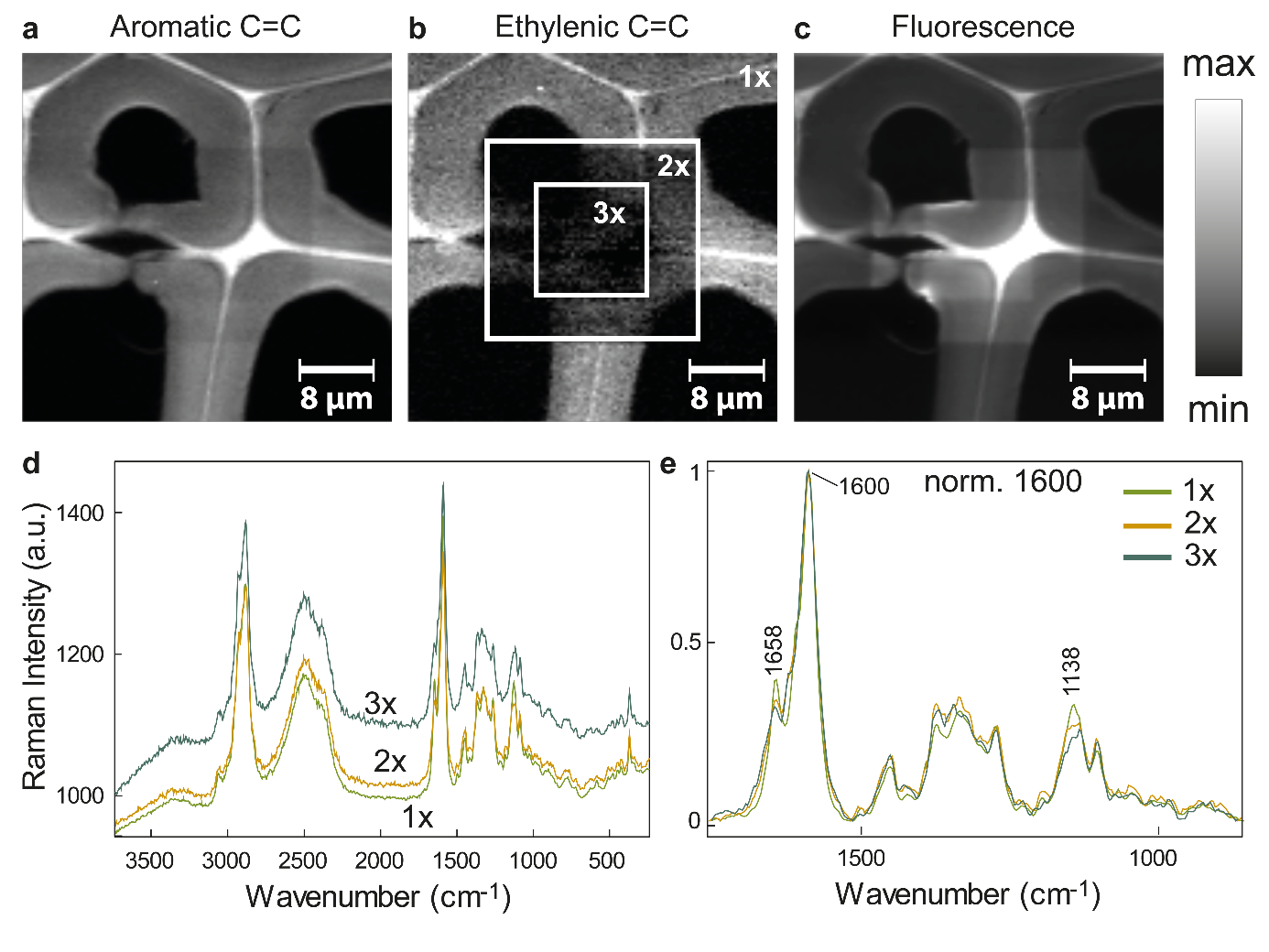


**Figure S1. Repeated laser exposure causes chemical changes during Raman imaging of pine sapwood (*Pinus sylvestris*). (a)** Integration over the main aromatic C=C stretching of lignin, **(b)** the ethylenic C=C stretching and the **(c)** fluorescent background. The inserted frames depict the successive concentric number of Raman images taken (“1x” means that the framed area was measured once, “2x” twice and so on). The aromatic C=C remains stable over the three consecutive measurements. In contrast, the ethylenic C=C, attributed to coniferyl alcohol, decreases abruptly with increasing number of measurements. The fluorescence increases as shown by an increase of the background intensity at the area framed by “3x”. To compare the same area, the minimal common area measured (framed by “3x”) was used as a mask to average the three consecutive Raman images. The raw (not pre-preprocessed) Raman average spectra of the three consecutive measurements can be found in **(d)**. The spectra were normalized over the aromatic C=C ring stretching for better visualization in **(e)**.


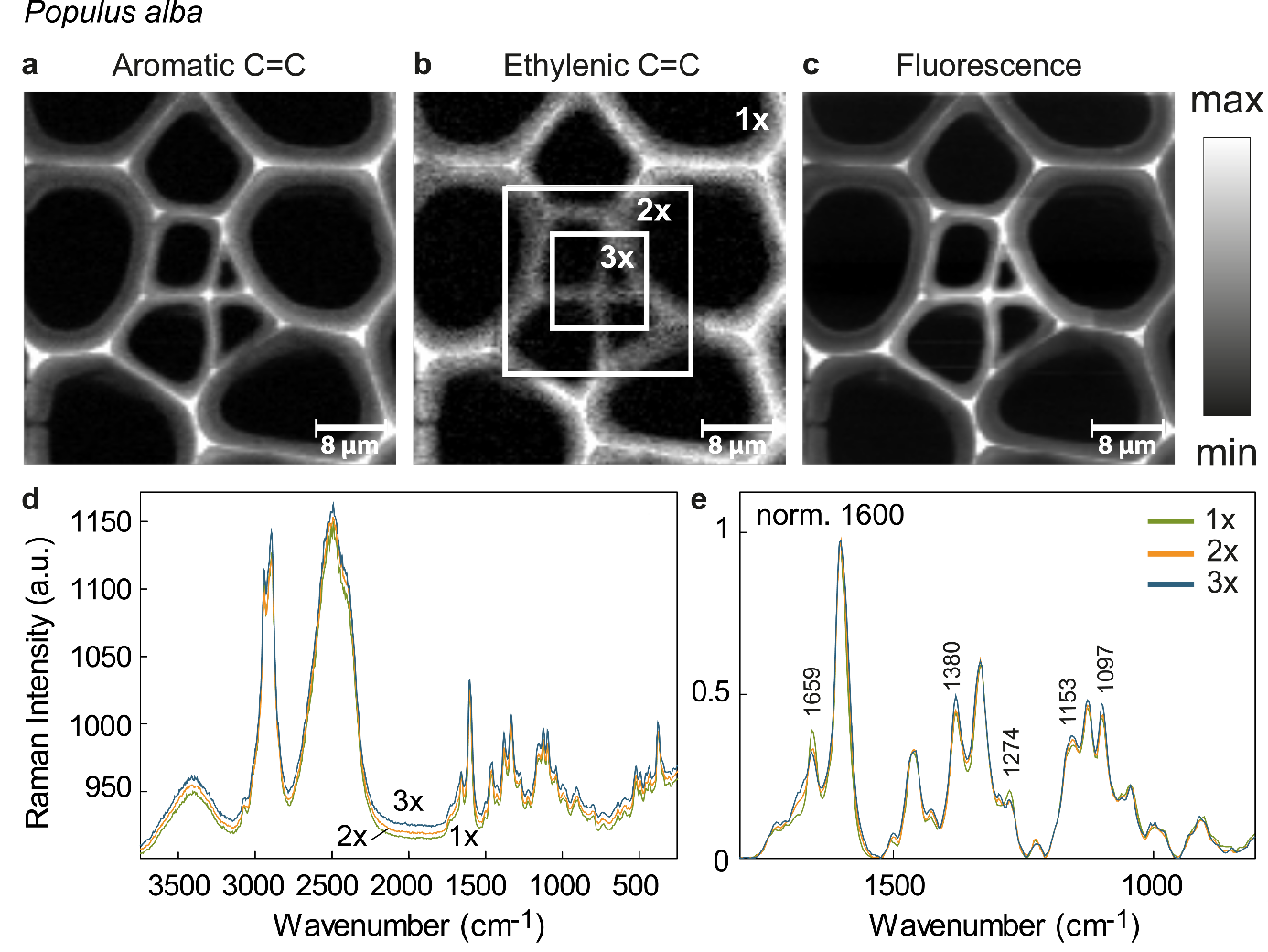


**Figure S2. Repeated laser exposure causes chemical changes during Raman imaging of poplar (*Populus alba*). (a)** Integration over the main aromatic C=C stretching of lignin, **(b)** the ethylenic C=C stretching and the **(c)** fluorescent background. The inserted frames depict the successive concentric number of Raman images taken (“1x” means that the framed area was measured once, “2x” twice and so on). The aromatic C=C remains stable over the three consecutive measurements. In contrast, the ethylenic C=C, attributed to coniferyl alcohol, decreases abruptly with increasing number of measurements. The fluorescence did not increase enormously as compared to pine or spruce. To compare the same area, the minimal common area measured (framed by “3x”) was used as a mask to average the three consecutive Raman images. The raw (not pre-preprocessed) Raman average spectra of the three consecutive measurements can be found in **(d)**. The spectra were normalized over the aromatic C=C ring stretching for better visualization in **(e).**


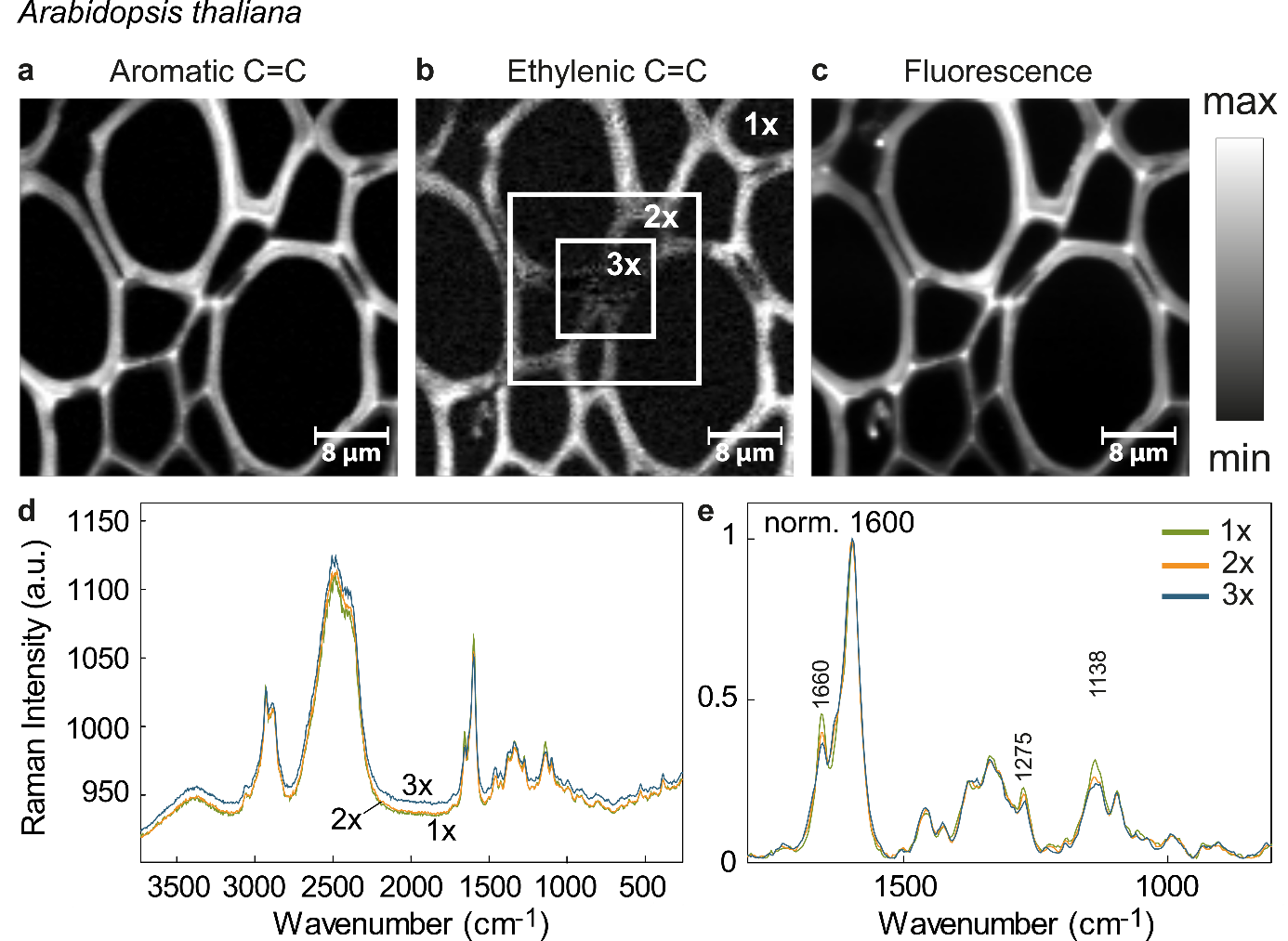


**Figure S3. Repeated laser exposure causes chemical changes during Raman imaging of *Arabidopsis thaliana*. (a)** Integration over the main aromatic C=C stretching of lignin, **(b)** the ethylenic C=C stretching and the **(c)** fluorescent background. The inserted frames depict the successive concentric number of Raman images taken (“1x” means that the framed area was measured once, “2x” twice and so on). The aromatic C=C remains stable over the three consecutive measurements. In contrast, the ethylenic C=C, attributed to coniferyl alcohol, decreases abruptly with increasing number of measurements. The fluorescence did not increase enormously as compared to pine or spruce. To compare the same area, the minimal common area measured (framed by “3x”) was used as a mask to average the three consecutive Raman images. The raw (not pre-preprocessed) Raman average spectra of the three consecutive measurements can be found in **(d)**. The spectra were normalized over the aromatic C=C ring stretching for better visualization in **(e)**.

| **a** | **Laser power**  **(mW)** | **Laser power corrected (mW)** | **Laser intensity**  **(W/cm^2^)** | **Exposure time**  **(s)** | **Energy density**  **(kJ/cm^2^)** |
| --- | --- | --- | --- | --- | --- |
|  | 10 | 9.5 | 4.6x10^5^ | 0.04 | 20.1 |
|  |  |  |  | 0.13 | 61.4 |
|  |  |  |  | 1.03 | 474.9 |
|  | 20 | 19 | 9.2x10^5^ | 0.04 | 40.2 |
|  |  |  |  | 0.13 | 122.9 |
|  |  |  |  | 1.03 | 949.8 |
|  | 30 | 28.5 | 1.4x10^6^ | 0.04 | 60.2 |
|  |  |  |  | 0.13 | 184.3 |
|  |  |  |  | 1.03 | 1424.7 |

|  |  |  |  |  |  |
| --- | --- | --- | --- | --- | --- |
| **b** | **Laser power**  **(mW)** | **Laser power corrected (mW)** | **Laser intensity**  **(W/cm^2^)** | **Exposure time**  **(s)** | **Energy density**  **(kJ/cm^2^)** |
|  | 10 | 7.3 | 4.3x10^6^ | 0.04 | 189.0 |
|  |  |  |  | 0.13 | 578.2 |
|  |  |  |  | 1.03 | 4470.3 |
|  | 20 | 14.6 | 8.6x10^7^ | 0.04 | 378.1 |
|  |  |  |  | 0.13 | 1156.5 |
|  |  |  |  | 1.03 | 8940.7 |
|  | 30 | 21.9 | 1.3x10^8^ | 0.04 | 567.1 |
|  |  |  |  | 0.13 | 1734.7 |
|  |  |  |  | 1.03 | 13411.0 |

**Table S4. Laser intensity and energy density for different laser power, exposure time at λ_ex_=532 nm** using an optical objective of 20x **(a)** (NA 0.4, transmittance of 95%) and 100x **(b)** (immersion oil, NA 1.4, transmittance of 73%) during Raman imaging. Depending on the laser power applied before the objective aperture, the exposure time used, and the objective used, the intensity of the laser and the irradiation dose on the sample changes. The energy on the sample was calculated taking into account the irradiation area *i.e.* laser spot area after passing through the objective. The laser spot was estimated based on the Rayleigh criterion (**equation (1)**). The resolution “r_xy_” was used as the radius for the calculation of the laser spot, modelled as the area of a circle (π* r_xy_^2^).


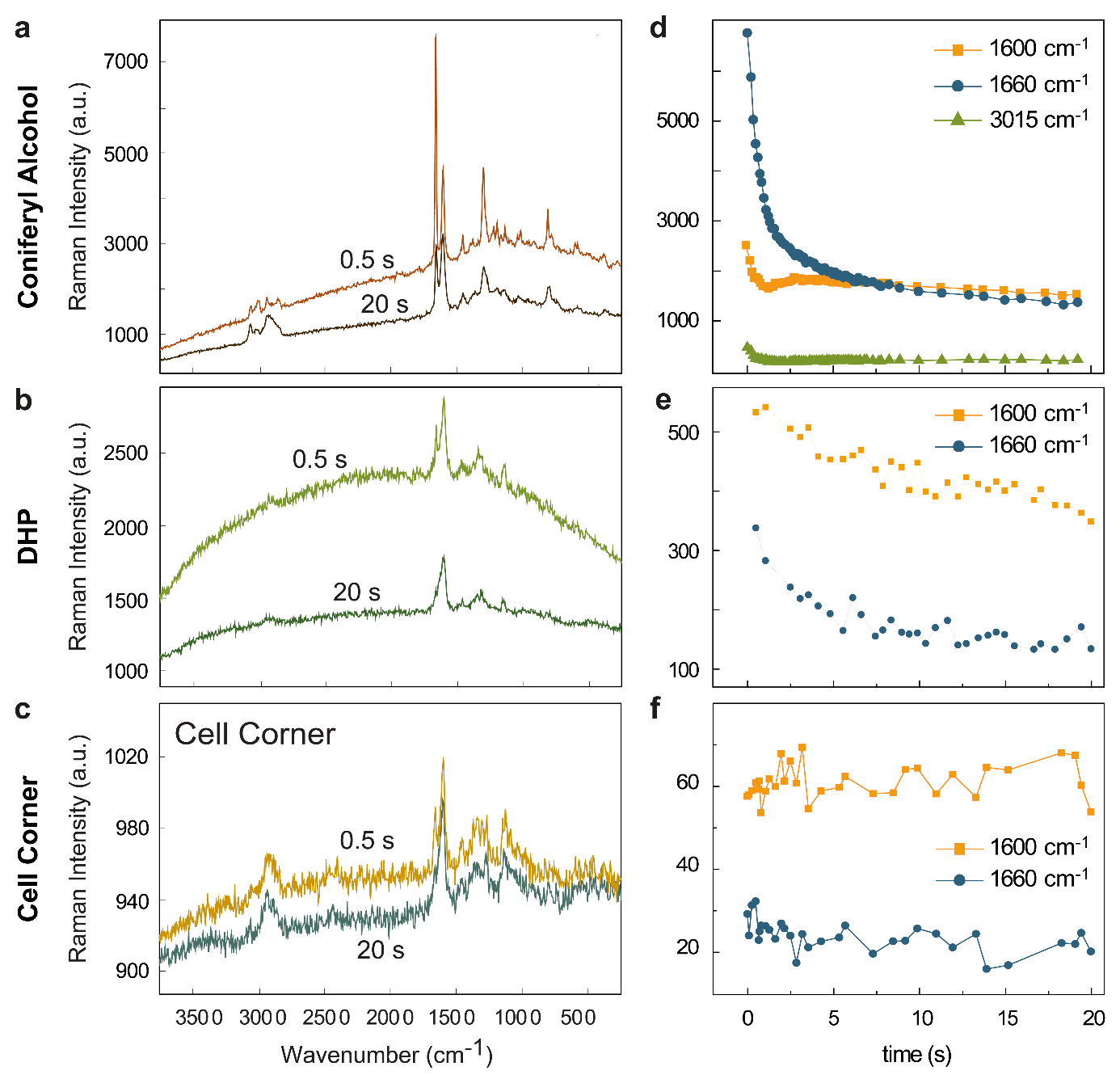


**Figure S5. (a-c) Examples of Raman spectra of reference compounds at t_0_ and t_0_+2.5 s exposure times for coniferyl alcohol, DHP and Spruce lignin (cell corner), respectively. (d-e) Band intensities of coniferyl alcohol, DHP and lignin of Spruce wood over time from which the ratios in Fig.3b-d were calculated.** For coniferyl alcohol, the intensity of the aromatic C=C at 1600 cm^-1^, the ethylenic C=C at 1660 cm^-1^ and the ethylenic CH=C at 3015 cm^-1^ decreased exponentially over time. For DHP and lignin of Spruce the 1600 and 1600 cm^-1^ decayed as well.


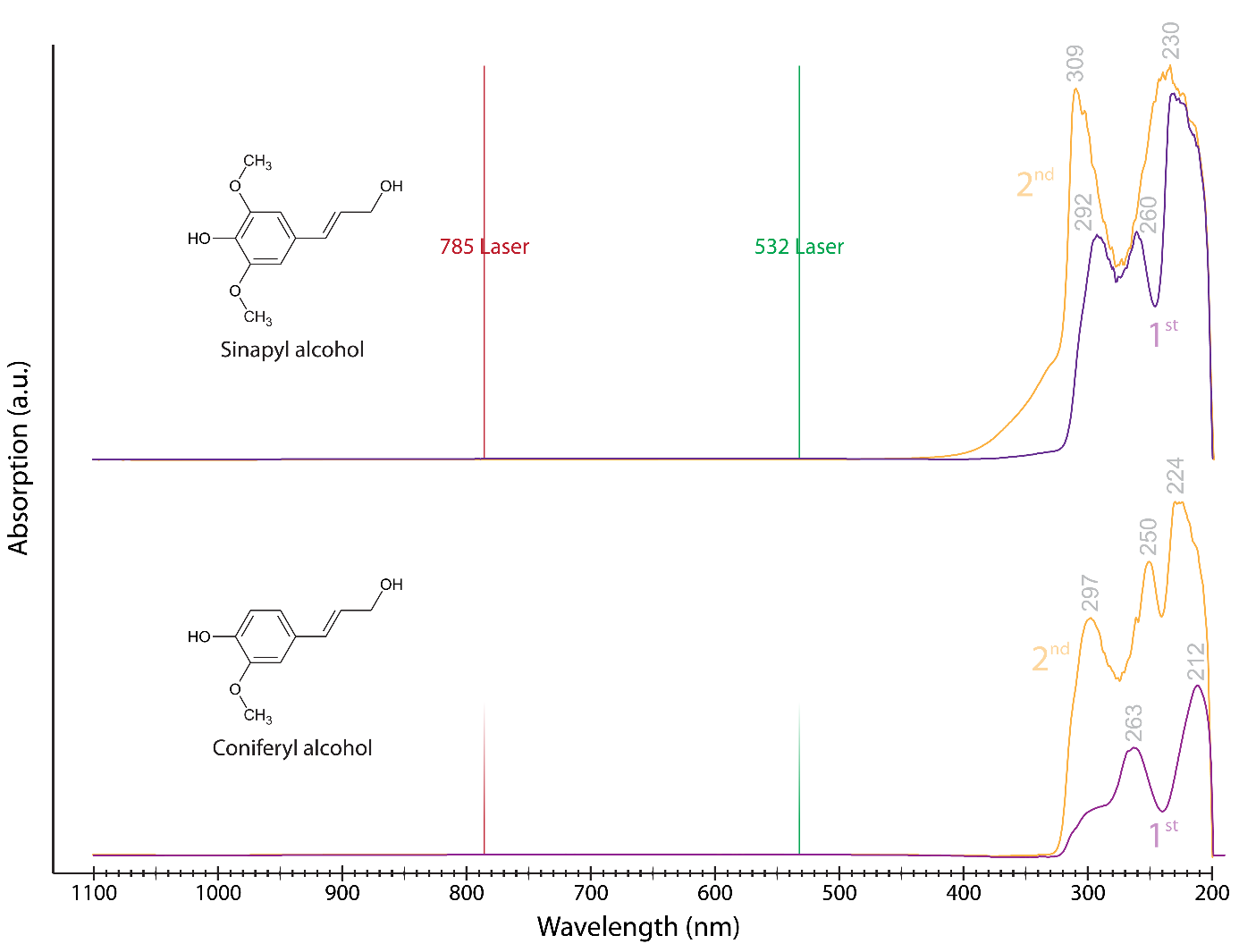


**Figure S6. UV-Vis absorption spectrum of Coniferyl and Sinapyl alcohol.** Ethanol was used as solvent. Displayed are two consecutive measurements, respectively. The laser wavelengths used in our experiments are also indicated.

**Supplementary Method S7**

All chemicals were purchased from Sigma-Aldrich and were used as received.

^1^H Magnetic Resonance (NMR) Measurements were collected on a Bruker DPX operating at 300 MHz in CDCl_3_ as solvent using TMS as an internal standard.

**Synthesis of Sinapyl alcohol:** To a solution of Sinapaldehyde (268.0 mg, 1.29 mmol) in dry methanol (15 mL) sodium borohydride (77.5 mg, 2.0 mmol) was slowly added at O°C. The reaction mixture was stirred for 15 min at this temperature and let warm to room temperature overnight. The reaction was quenched with 10 mL water. The solvent was evaporated and the residue was dissolved in 20 mL dichloromethane. Followed by an extraction twice with water (20 mL) and once with conc. sodium chloride solution (20 mL). The organic phase was dried over sodium sulphate, filtrated and dried *in vacuum* (yield: 185 mg, 0.88 mmol, 68%).

^1^H NMR (300 MHz, CDCl_3_) δ 3.90 (s, 6H), 4.31 (t, 2H, ^3^J_HH_ = 4.7 Hz), 6.23 (dt, 1H, ^3^J_HH_ = 15.8 Hz, ^3^J_HH_ = 5.9 Hz), 6.52 (dt, 1H, ^3^J_HH_ = 15.7Hz, ^4^J_HH_ = 1.4), 6.63 (m, 3H, ArH).

**Synthesis of Cinnamyl alcohol:** A solution of Cinnamaldehyde (178 mg, 1.35 mmol) in dry methanol (5 mL) was cooled with ice to 0°C. After the addition of sodium borohydride (71.7 mg, 1.9 mmol) the reaction mixture was stirred at 0°C for 15 min and then let warm to room temperature overnight. The reaction was quenched with water (10 mL). The solvent was evaporated and dissolved in dichloromethane (20 mL). The product was extracted twice with water (20 mL) and once with conc. sodium chloride solution. It was dried over sodium sulphate, filtrated and dried *in vacuum* (yield: 179 mg, 1.33, 99%)

^1^H NMR (300 MHz, CDCl_3_) δ 2.18 (s, 1H, -OH), 3.90 (s, 6H), 4.31 (t, 2H, ^3^J_HH_ = 4.7 Hz), 6.38 (dt, 1H, ^3^J_HH_ = 15.8 Hz, ^3^J_HH_ = 5.7 Hz), 6.52 (d, 1H, ^3^J_HH_ = 16.0 Hz), 7.33 (m, 5H, ArH).
